# Chicken cyclophilin A is an inhibitory factor to influenza virus replication

**DOI:** 10.1186/1743-422X-7-372

**Published:** 2010-12-30

**Authors:** Chongfeng Xu, Shanshan Meng, Xiaoling Liu, Lei Sun, Wenjun Liu

**Affiliations:** 1 Graduate University of Chinese Academy of Sciences, Beijing 100101, China; 2 CAS Key Laboratory of Pathogenic Microbiology and Immunology, Institute of Microbiology, Chinese Academy of Sciences, Beijing 100101, China; 3 China-Japan Joint Laboratory of Molecular Immunology and Molecular Microbiology, Institute of Microbiology, Chinese Academy of Sciences, Beijing 100101, China

## Abstract

**Background:**

The importance of enhancing influenza resistance in domestic flocks is quite clear both scientifically and economically. Chicken is very susceptible to influenza virus. It has been reported that human cellular cyclophilin A (CypA) impaired influenza virus infection in 293T cells. Whether chicken CypA (chCypA) inhibits influenza virus replication is not known. The molecular mechanism of resistance in chicken to influenza virus remains to be studied.

**Results:**

The chCypA gene was isolated and characterized in the present study. It contained an ORF of 498 bp encoding a polypeptide of 165 amino acids with an estimated molecular mass of 17.8 kDa sharing high identity with mammalian CypA genes. The chCypA demonstrated an anti-influenza activity as expected. ChCypA protein was shown to be able to specifically interact with influenza virus M1 protein. Cell susceptibility to influenza virus was reduced by over-expression of chCypA in CEF cells. The production of recombinant influenza virus A/WSN/33 reduced to one third in chCypA expressing cells comparing to chCypA absent cells. ChCypA was widely distributed in a variety of chicken tissues. It localized in cytoplasm of chicken embryo fibroblast (CEF) cells. Avian influenza virus infection induced its translocation from cytoplasm into nucleus. ChCypA expression was not significantly up-regulated by avian influenza virus infection. The present study indicated that chCypA was an inhibitory protein to influenza virus replication, suggesting a role as an intrinsic immunity factor against influenza virus infection.

**Conclusion:**

The present data demonstrates that chCypA possesses anti-influenza virus activity which allows the consideration of genetic improvement for resistance to influenza virus in chickens.

## Background

In chicken, influenza A virus infection causes a wide spectrum of symptoms, ranging from mild illness to a highly contagious and rapidly fatal disease resulting in severe epidemics, which not only cause great economic loss for poultry industry but also pose threat to public health.

It is hypothesized that the major cell determinant of resistance to influenza virus is absence of the counter receptors on cell surface. Therefore the viral haemagglutinin is not able to access such cells to initiate first step of infection. However, it has been reported recently that both SAα2, 6-Gal and SAα2, 3-Gal receptors are present in many organs of birds, pigs and humans [1-4]. The susceptibility to influenza virus of different host species varies greatly suggesting existence of inhibitory mechanisms beyond the receptor-virus interaction. Multiple layers of defence systems are present in host cells to either block entrance or inhibit viral replication during the course of infection. In the present study chicken cellular protein chCypA was proven to be such a host factor inhibitory to influenza virus infection and replication. Our study suggests the ubiquitous protein serves as a defensive mechanism against influenza virus infection.

It has been shown that CypA is incorporated into influenza virus virion [5] and the expression of CypA is up-regulated upon infection by avian influenza virus in a human gastric carcinoma cell line [6]. CypA exhibits an inhibitory activity to influenza virus replication in the early stage of infection by interfering newly synthesized M1 protein translocation into nucleus [7].

Cyclophilin A is a multifunctional protein which is the major cytosolic binding protein of the immunosuppressive drug cyclosporin A. CypA has a chaperone-like activity of peptidylprolyl cis-trans isomerase, which may play important roles in protein folding, trafficking, assembly, immune-modulator and cell signalling. CypA also involves in pathogenesis in several diseases including cancer, cardiovascular disease and viral infection. Several studies suggest an essential or inhibitory role for CypA in the replication of several viruses including human immunodeficiency virus type 1, vesicular stomatitis virus, vaccinia virus, hepatitis C virus and human papillomavirus type 1[8-17].

To determine whether chCypA possesses the similar anti-influenza activity as human CypA, chCypA was isolated and characterized and the relevance of chCypA to influenza virus infection was revealed. The discovery that chCypA inhibited influenza virus replication may lead to consideration of genetic improvement in chicken for resistance to influenza virus. Comprehensive knowledge of host restriction factors to influenza virus infection could provide valuable insight into the molecular mechanisms of viral replication and cellular defensive response to virus infection.

## Results

### cDNA cloning, sequence analysis of chCypA and functional sites prediction

**Figure 1:**
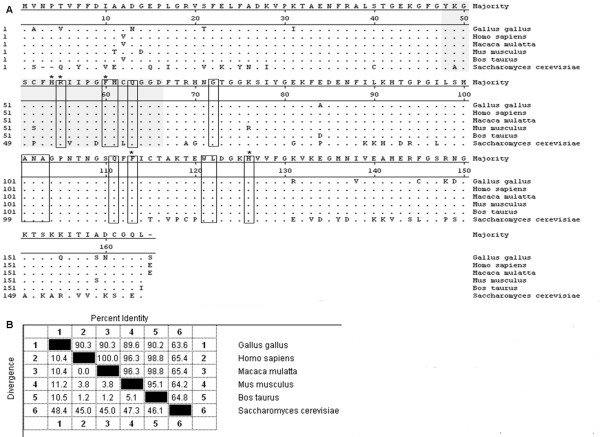
**Alignment of deduced amino acid sequence of chCypA homology from different species**. A, Amino acid residues aligned by the CLUSTALW program. The residues that match the consensus exactly were shown with ".", the Cyp-type peptidyl-prolyl cis-trans isomerase signatures and the well conserved CsA binding sites were shadowed; the amino acids necessary for the peptidylprolyl cis-trans isomerase activity were marked with"*"; thirteen CsA binding residues were boxed. B, Homology identity between chCypA and other species cyclophilin A. GenBank accession numbers are: P62937 (human), P62940 (rhesus monkey), P17742 (mouse), P62935 (cattle), P14832 (baker's yeast).

The full length ORF of chCypA was composed of 498 bp [GenBank: GQ849480] encoding a polypeptide of 165 amino acids with a predicted molecular mass of 17.8 kDa. The isoelectric point of chCypA predicted with DNAStar program was 8.07. The deduced amino acid sequences shared homology with those of human CypA and bovine CypA were 90.3% and 90.2% respectively, the homology between the mammalian CypAs is over 96% (Figure 1B).

Although chCypA shared 90.3% amino acid homology with human CypA, polyclonal antibodies against human CypA could not detect endogenous chCypA (data not shown), which suggested an antigenic difference exist between human CypA and chicken CypA. Results analysed by DNAStar protean software suggested the high antigenicity region was mostly localized in amino acid residues from 147 to 155 within chCypA where showing high surface probability and high antigenic index. The different antigenicity could be explained by the fact that most different amino acids between chCypA and human CypA were also localized within this region (Figure 1A).

It has been reported that chCypA with Mr18 kDa and pI 8.2 [18], approximate to Mr17.8 kDa and pI 8.07 which we predicted from ORF sequence with DNAStar program. Rabbit polyclonal antibodies against chCypA generated with purified hexahistidine-tagged chCypA (His-chCypA) could detect endogenous 18 kDa protein confirming the gene isolated was indeed chicken cyclophilin A. There were two amino acid residues (Pro12Ala, Thr20Val) different from our gene in partial chCypA reported by Caroni [19]. The peptidylprolyl cis-trans isomerase active site (residues His54, Arg55, Phe60, Gln111, Phe113, Trp121) and thirteen CsA contact residues (Arg55, Phe60, Met61, Gln 63, Gly72, Ala101, Asn102, Ala103, Gln111, Phe113, Trp121, Leu122 and His126) were exactly same among the sequences aligned (Figure 1A).

### ChCypA interacted with influenza virus M1 protein

**Figure 2:**
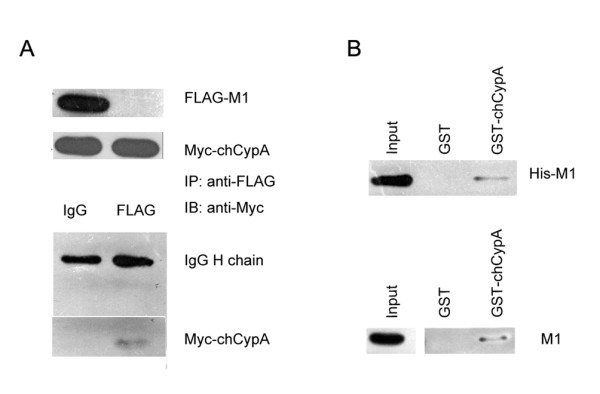
**ChCypA interacted with M1 protein of influenza virus *in vitro *and *in vivo***. A. Co-immunoprecipitation of M1 and chCypA. Input shows 1/10 of the total proteins included in each binding reaction. Lane 1, pcDNA3-FLAG-M1 and pCMV-Myc-chCypA plasmids were simultaneously transfected into 293T cells. Lane 2, pCMV-Myc-chCypA plasmid was transfected in 293T cells. 48 h after transfection, the cells were lysed in Hepes buffer prepared for co-immunoprecipitation. Co-immunoprecipitation was performed using anti-FLAG monoclonal antibody, and the proteins immunoprecipitated (IP) were detected with an anti c-Myc monoclonal antibody. B. GST pull-down assay was used to detect the interaction of influenza A virus M1 protein and chCypA *in vitro*. His-M1 fusion protein (1 mg) was incubated with an equal amount of GST alone or GST-chCypA bound to glutathione-sepharose 4B beads. After washing extensively, the His-M1 bound to the beads was extracted and analyzed by Western blot with anti-His antibodies. The CEF cell lysates infected by A/Chicken/Liaoning/1/00 (H9N2) were incubated with GST alone (lane GST) or GST-chCypA (lane GST-chCypA) bound to glutathione-sepharose 4B beads. After washing extensively, the proteins bound to the beads were detected by Western blot analysis using anti-M1 monoclonal antibodies. Input shows 1/10 of total M1 proteins in each binding reaction.

To detect whether chCypA directly interacted with M1 protein, GST pull-down assay and co-immunoprecipitation were performed. The specific interaction of M1 and chCypA *in vivo *was determined by co-immunoprecipitation with proteins transient expressed in 293T cell. As shown in Figure 2A, Myc-chCypA could be co-immunoprecipited with Flag-M1 by anti-Flag monoclonal antibody. In GST pull-down assay, His-M1 fusion protein or CEF cell lysate infected with avian influenza virus A/Chicken/Liaoning/1/00(H9N2) was incubated with an equal amount of GST alone or GST-chCypA recombinant protein bound to glutathionesepharose 4B beads. After washing extensively, the His-M1 or M1 protein bound to the beads was extracted and analysed by Western blot with anti-His-tag or anti-M1 monoclonal antibody. As shown in Figure 2B, the M1 protein was associated with GST-chCypA, but not GST alone. GST pull-down and co-immunoprecipitation assays showed that chCypA as its homology human CypA could specifically interact with M1 *in vitro *and *in vivo*.

### The production of recombinant A/WSN/33 virus was reduced in over-expressing chCypA cells

**Figure 3:**
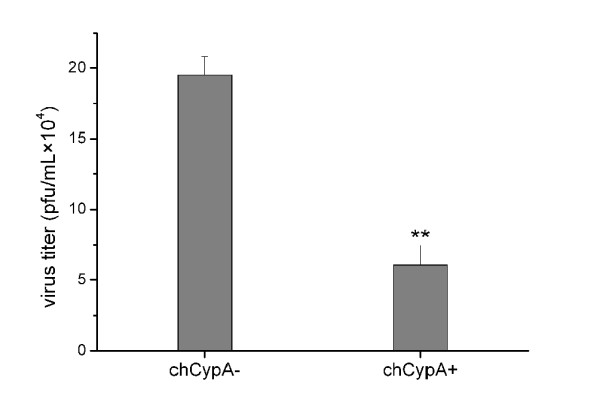
**The effect of chCypA on A/WSN/33 virus production**. The inhibitory effect of chCypA on influenza virus replication was tested by using reverse genetic system of A/WSN/33 [49]. In brief, both a mixture of expression plasmids of PB1, PB2, PA and NP proteins and a whole set of virus RNA expression plasmids were transfected into 293T/CypA- cells in the presence or absence of chCypA expression plasmid pCMV-Myc-chCypA, pCMV-Myc was transfected as control. The supernatant was harvested 48 hpt and titrated on MDCK cells using plaque assay. Significant differences across control were indicated with two asterisks at P < 0.01.

12 plasmids reverse genetic system were transfected into 293T/CypA- cell with chCypA expressing plasmid pCMV-Myc-chCypA. PCMV-Myc was used as vector control. In chCypA over-expressing 293T/CypA- cells, the packed A/WSN/33 virus particle production was reduced to one-third of that in pCMV-Myc transfected cells (p < 0.01) (Figure 3). This result suggested that chCypA inhibited the virus replication in 293T/CypA- cells.

### ChCypA reduced cell susceptibility to A/Chicken/Liaoning/1/00(H9N2) infection

**Figure 4:**
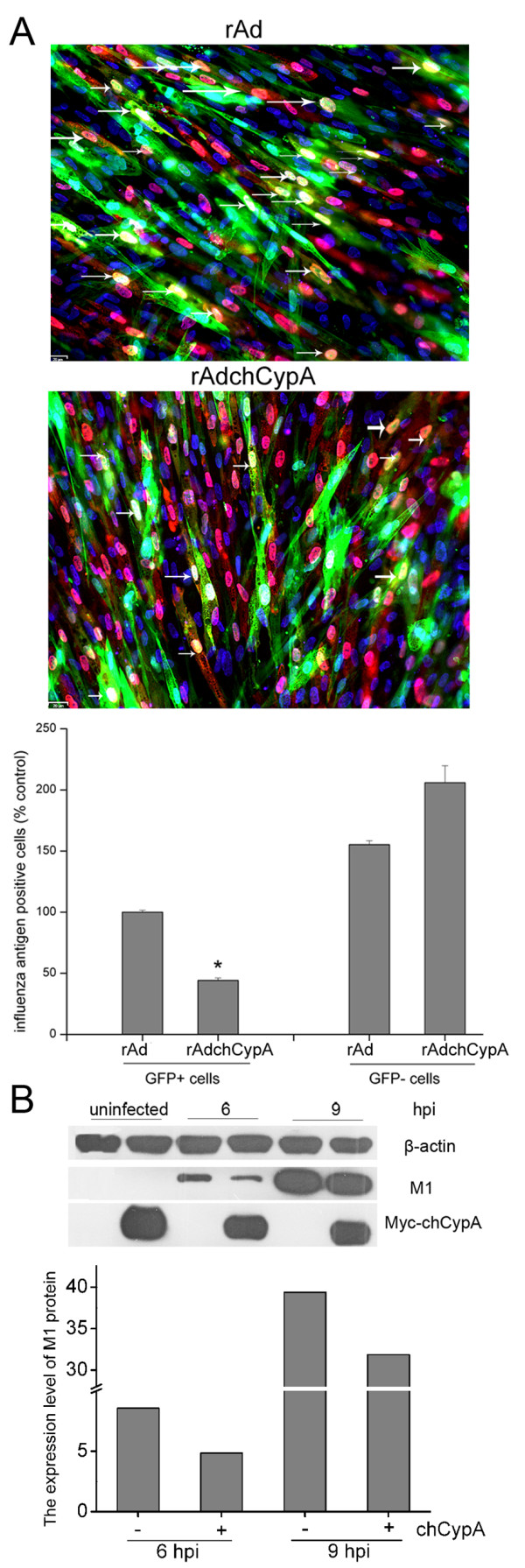
**The effect of ChCypA on avian influenza virus infectivity**. A. CEF cells were infected with chCypA-expressing recombinant adenovirus rAdchCypA (or rAd as control) and infected after 30 h with A/Chicken/Liaoning/1/00 (H9N2) (MOI = 1), 4 h post influenza virus infection, cells were stained for NP (red). The percentage of influenza virus antigen positive cells in GFP positive population relative to that for the rAd control group was shown below. Scale bar: 20 μm. *P < 0.05. B. ChCypA inhibited expression of M1 protein. 293T/CypA- cells were transfected with pCMV-Myc or pCMV-Myc-chCypA, 24 h post transfection, cells were infected with A/Chicken/Liaoning/1/00 (H9N2) (MOI = 0.01). 6 h and 9 h post infection, cells were harvested. Proteins were detected by Western blot analysis using anti-β-actin, M1 and c-Myc monoclonal antibodies as indicated. The relative expression level of M1 protein was indicated by densitometry of M1 band on Western blot X film. The densitometry of target protein band was analyzed with Photoshop program.

Recombinant adenovirus carrying chCypA gene named rAdchCypA was generated to deliver the chCypA gene into CEF cells. Empty adenovirus rAd was also generated as vector control. Thirty hours post recombinant adenovirus infection, CEF cells were infected with A/Chicken/Liaoning/1/00(H9N2) at MOI = 1. 4 hours post infection (hpi), cells were detected by immunostaining with anti-influenza NP polyclonal antibody and TRITC-conjugated secondary antibody. GFP positive cells represented recombinant adenovirus infected cells were counted under the Fluorescence Microscopy. Data was shown in Figure 4A. In chCypA over-expression group, there was an approximately three-fold reduction (p < 0.05) in the proportion of the NP positive cells relative to rAd infected CEF cells. In recombinant adenovirus uninfected subpopulations, i.e., GFP-negative cells, the antigen staining was seemly enhanced relative to rAd infected control (Figure 4A, panel below). Nevertheless, no statistic significance was found (p = 0.32) comparing those in rAd infected with rAdchCypA infected groups. This confirmed that the inhibition of viral infectivity was specific to the chCypA over-expressed cells. The cell susceptibility to influenza virus was reduced by over-expression of chCypA was further confirmed by the results in which over-expression of chCypA on 293T/CypA- cells could inhibit influenza virus M1 protein expression (Figure 4B).

### ChCypA was widely distributed in all tissues detected

**Figure 5:**
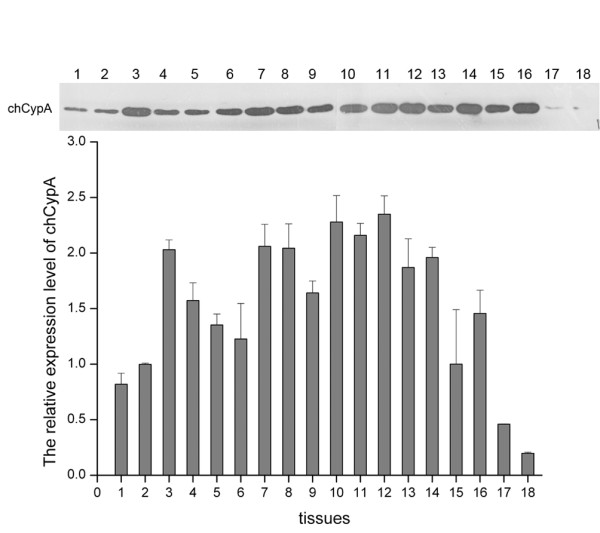
**The distribution of chCypA in different tissues**. Eighteen tissues were extracted from 21-day-old SPF chickens and relative protein level of chCypA was determined by Western blot and relative densitometry analysis carried out with Photoshop program. 2 μg tissues extracts were loaded into SDS-PAGE. 1, heart 2, liver, 3, spleen 4, lung 5, kidney 6, pancreas 7, brusa of Fabricius 8, esophagus 9, duodenum 10, thymus 11, cerebrum 12, cerebella 13, glandular stomach 14, gizzard 15, muscle 16, trachea 17, ovary 18, blood. Tissues of three chickens had been extracted, and the representative data is shown.

Tissues of three 21-day-old SPF chickens were extracted and homogenized. 2 μg extractions of tissues were loaded to SDS-PAGE and detected with anti-chCypA polyclonal antibody by Western blot. Abundant chCypA was widely distributed in all the tissues tested (Figure 5). The relative expression level of chCypA in different tissues was analyzed by comparing the intensity of bands on X-ray films of Western blot with Photoshop software. Intensity of chCypA in variant tissues was normalized to density of bands for liver on X-ray films. Results of densitometry analysis indicated the chCypA expression levels in spleen, bursa of Fabricius, thymus and cerebella were much higher than levels expressed in other tissues.

### The temporal expression of chCypA was not significantly up-regulated upon avian influenza virus infection

**Figure 6:**
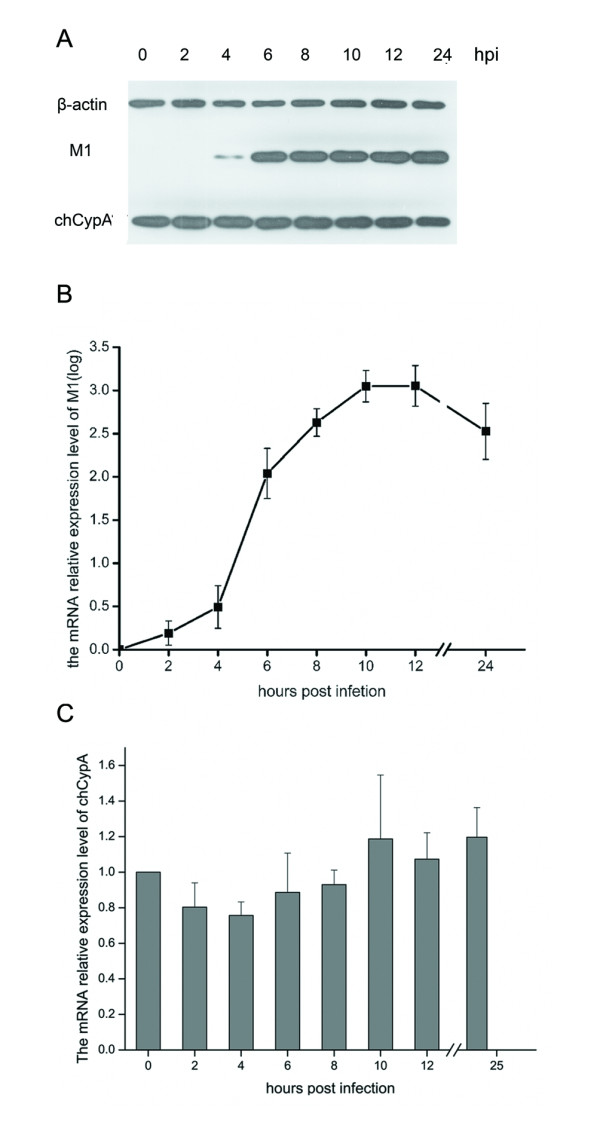
**The temporal expression level of chCypA in CEF cells after influenza virus infection**. A. The temporal expression of chCypA in CEF cells after influenza virus infection was measured with Western blot analysis (MOI = 0.1). B. The temporal mRNA relative expression level of influenza virus M1 was measured by qRT-PCR. C. Temporal relative expression of chCypA transcripts in CEF cells after influenza virus infection was measured by qRT-PCR. Relative gene expression was calculated with initial normalization to β-actin within each sample. Values are mean ± S.D. The relative expression value was averaged from three duplicates, each of which contains three independent samples.

To determine whether chCypA expression responds to influenza virus infection, the relative mRNA and protein expression levels of chCypA in CEF cells at 0, 2, 4, 6, 8, 10, 12, and 24 hpi were measured by quantitative real time PCR and Western blot respectively. There was no significant increase of expression of chCypA after A/Chicken/Liaoning/1/00(H9N2) infection (Figure 6).

### Intracellular localization of chCypA changed upon infection by A/Chicken/Liaoning/1/00(H9N2)

**Figure 7:**
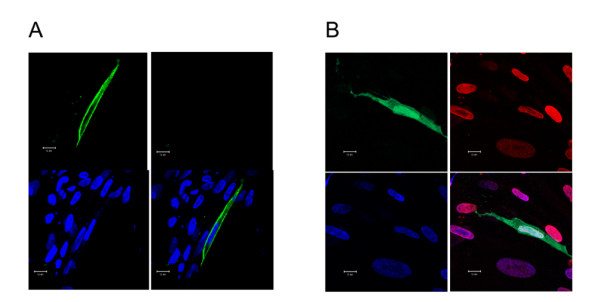
**Influenza virus infection induced nuclear localization of chCypA**. CEF cells were transfected with chCypA-expressing plasmid pCMV-Myc-chCypA, and infected with A/Chicken/Liaoning/1/00 (H9N2) (MOI = 5) or not (A) as control after 30 h transfection for 4 h, and were fixed and stained for Myc-chCypA (green) and M1 (red) protein, the nucleus was stained blue with DAPI. Scale bar: 10 μm.

Intracellular localization of the chCypA was determined by indirect immunofluorescence assay to assess the effect of influenza virus infection on chCypA translocation. CEF cells were trasfected with plasmid pCMV-Myc-chCypA, 30 h post-transfection (hpt), cells were fixed and immunostained with c-Myc polyclonal antibody. In non-infected control, as depicted in Figure 7A, Myc-chCypA was localized predominantly in cytoplasm. At 30 hpt, CEF cells were infected with avian influenza virus A/Chicken/Liaoning/1/00(H9N2) (MOI = 5). At 4 hpi, Myc-chCypA and M1 were stained for green and red respectively, as pictures shown in Figure 7B, a large proportion of Myc-chCypA translocated into nucleus.

## Discussion

It has been demonstrated that human CypA interacts with influenza virus M1 protein and impairs the early stage of the viral replication [7]. In the present study, chCypA was isolated and characterized. Results suggested that the chCypA was an inhibitory protein to influenza virus replication and infectivity. The expression of chCypA was not significantly up-regulated upon influenza virus infection. However, a fraction of chCypA present in cytoplasm was translocated into nucleus upon infection of avian influenza virus in CEF cells.

It is reported that over-expression of CypA inhibits influenza A virus infection [7]. One hypothesis is that different expression level of CypA in different tissues may contribute to the resistance to influenza A virus. In our study, the chCypA was detected in all tissues tested, as described in other species [20-24]. The concentration of CypA determined by CsA binding activity or other methods in different tissues varies in different animals [21-25]. The relative expression of chCypA in spleen, bursa of Fabricius, thymus and cerebella were much higher than which in other tissues. However, there is no obvious statistical relevance between the expression level of chCypA and tissue specific resistance to influenza virus.

It was reported that the expression of CypA in some animals was drastically up-regulated after lipopolysaccharides (LPS), Con A or bacteria challenged [21,23,24]. These findings suggest that CypA is a response protein to pathogen stimulation. The current study found that the expression of chCypA was not up-regulated by avian influenza virus infection tested by Western blot and quantitative real time RT-PCR. The latest findings suggested that CypA expression level was up-regulated in response to avian H9N2 virus infection in human cells [6]. This inconformity may be explained by the degree of host species adaptation to avian influenza virus infection. Our prediction is that human cells have not been well adaptive to H9N2 avian influenza virus invasion. It is possible that CypA might be up-regulated by this virus strain infection as a response to stress.

Viruses are obligate intracellular parasites that interact with host cell and use the host machinery for their replication. On the other hand, there exist host factors that restrict viral replication. The current perspectives about host defence system, mediated by some of these restriction factors designated as intrinsic immunity, is distinguished from conventional innate and adaptive immunity system as has been reviewed by Bieniaz and Takeuchi [26-28]. Recent discoveries have revealed previously unappreciated complexity with which influenza virus interact with their hosts [29]. In particular, we have become aware that mammals and birds are also equipped with genes encoding so-called "restriction factors", that provide considerable resistance to influenza virus infection. Heat shock cognate protein 70 (Hsc70) inhibits the nuclear export of M1 and NP [30]. Ebp1, ErbB3-binding protein specifically interacts with PB1 and interferes with RNA polymerase activity [31]. Interferon induced proteins mouse Mx1 and human MxA suppress influenza virus transcription by interacting with PB2 and NP [32]. ISG15 inhibits influenza A virus gene expression and replication [33]. Viperin inhibits influenza virus release by perturbing lipid rafts [34]. In the present report, we provide evidence that the ubiquitous protein CypA in chicken serves as a constitutively expressed inhibitor to influenza virus replication. Therefore we propose host factor chCypA is involved in intrinsic immunity against influenza virus infection. The inhibition to influenza virus of chCypA is depended on the interaction with M1 protein. M1 protein is well conserved among influenza A viruses, so it is believed that chCypA possesses inhibitory effect on broad spectrum of influenza A viruses including highly pathogenic avian influenza virus (HPAIV). Interestingly, CypA and Trim5 were also found to resistant to HIV-1 infection [15,35-37]. On the other hand, CypA is required for HCV replication [12,38]. It is noteworthy that the same host protein can play a different role in life cycle of different viruses.

ChCypA displayed an essentially cytoplasm localization and nuclear translocation upon influenza virus infection as evidenced by indirect immunofluorescence. Other studies also show that CypA can change location between different cellular apartments. It is reported that CypA phosphorylation and nuclear translocation can be induced by ligand stimulation of chemokine receptor CXCR4 [39]. Vaccinia virus infection can cause CypA redistribution to viral factories [10]. We have demonstrated here that chCypA is involved in influenza virus infection. However, little is known about the biological significance of nuclear translocation of chCypA.

It has been reported that CypA is a proinflammatory factor [40], implicating its potential role in cytokines induction and anti-influenza virus activity. IFN-β plays important roles in controlling viral infection in epithelial cells. Among members of peptidyl-prolyl isomerase superfamily, it is reported that cyclophilin B (CypB) plays a critical role in interferon regulatory factor-3 activation and virus induced production of IFN-β [41]. However, it is reported Pin1, another peptidyl-prolyl isomerase, as a negative regulator of interferon regulatory factor-3 dependent innate antiviral response [42]. The peptidyl-prolyl isomerase domain of CypB and Pin1 was required in regulation of IFN-β. Both chCypA and CypA are peptidyl-prolyl isomerases. So it is reasonable to assume that CypA might play a role in regulation of virus induced IFN-β production. However, it has been proved by our group that the inhibition of influenza virus by CypA is not depended on its isomerase activity [7]. It is believed that NS1 protein and polymerase complex of influenza A virus are potent blockers of activation of IFN-β, However, PB1-F2 exacerbates IFN-β expression [43-48]. The relevance between inhibitory activity of chCypA or CypA on influenza virus replication and the regulation potential in cytokines induction remains to be known.

The findings described in our study indicated that chCypA exhibited an anti-influenza activity potentially by interacting with influenza virus M1 protein, and translocating into nucleus upon influenza infection. The precise mechanisms of anti-influenza function of chCypA remain to be explored. Further investigation of molecular mechanisms of how chCypA inhibits influenza virus replication may help us better understand its anti-infection potential. The discoveries made from this study will have some implications on a variety of scientific areas including genetic improvement for resistance to influenza virus infection, development of viral vectors for gene therapy and discovery of novel antiviral drug targets.

## Conclusions

This work demonstrates that chicken CypA is a well conserved and widely distributed protein and possesses an anti-influenza virus activity. Over-expression of chCypA reduced A/WSN/33 virus production to one-third of control, and inhibited influenza virus infectivity in CEF cells. ChCypA could translocate into nucleus from cytoplasm upon infection of influenza virus. Our data suggested that chCypA might be an intrinsic immunity factor to influenza virus infection.

## Materials and methods

### Cell lines, viruses, plasmids, and antibodies

The human embryonic kidney 293T cells, CypA gene knockout 293T cell 293T/CypA-, CEF, Madin-Darby canine kidney (MDCK) cells were maintained in Dulbecco's modified Eagle's medium (GIBICO) supplemented with 10% fetal bovine serum (GIBICO) 37°C and 5% CO_2_. Wild-type influenza A virus strain A/Chicken/Liaoning/1/00 (H9N2) was propagated in 9-day-old embryonic eggs, A/WSN/33 (H1N1) was rescued from cDNA [49] and titrated on MDCK cells with plaque assay. Recombinant adenoviruses were generated as described by Luo [50], chCypA gene was subcloned into shuttle vector pAdTrack-CMV, the resultant plasmid was linearized by digesting with restriction endonuclease PmeI and subsequently transformed into competent AdEasier cells BJ5183, derivatives containing the adenoviral backbone plasmid pAdEasy-1. Recombinants were selected for kanamycin resistance and recombination was confirmed by restriction endonuclease analyses. The confirmed recombinant adenovirus plasmids were digested with PacI and transfected into 293A cells to generate recombinant adenoviruses rAdchCypA carrying chCypA gene. Expression of chCypA with rAdchCypA was verified by infection 293T/CypA- and detected by Western blot with His-chCypA polyclonal antibodies. Rabbit polyclonal antibodies against chCypA were generated by immunization of 2-month-old female rabbits with 250 μg of purified hexahistidine-tagged chCypA (His-chCypA) in Freund's complete adjuvant; the generation of antibodies was boosted three times by immunization with 150 μg of the protein at 2 week intervals. Mouse anti-M1 monoclonal antibody was prepared as described previously [7]. Anti-β-actin (Proteintech group, Catalog No: 60008-1-Ig). C-Myc (9E10) antibodies were purchased from Santa Cruz Biotechnology. Mouse anti-FLAG (M2) antibody and anti-c-Myc polyclonal antibody were purchased from Sigma. TRITC-conjugated anti-mouse IgG and FITC-conjugated anti-rabbit IgG were purchased from Zhongshan Golden Bridge Biotechnology, Beijing, China.

### Isolation of full length chCypA ORF

Total RNA of 10-days SPF chicken embryo brain was extracted using TRIzol (Invitrogen) reagent following the protocol of manufacturer, and dissolved in DEPC treated water and stored at -80°C. The First strand cDNA of chCypA gene was synthesized by reverse transcriptase (RT) using SuperScript III RT (Invitrogen) and oligo (dT)12-18 as primer. The complete chCypA ORF was amplified with PCR from first strand cDNA with rTaq polymerase (TAKARA, Japan) and primers chCypA-F: 5'ATGAATTCGGATGGCCAACCCCGTCG-3' and chCypA-R: 5'TGCTCGAGTTACGAGAGCTGCCCGC-3'. The PCR amplified chCypA genes were cloned into pCMV-Myc, pET- 30a, pGEX-4t-2 plasmids.

### GST pull-down assays and Co-immunoprecipitation

GST pull-down and co-immunoprecipitation assays were performed as described previously [7]. Briefly, *Escherichia coli *BL21 (DE3)/pGEX-chCypA, was cultured to mid-log phase at 37°C, isopropyl-1-thio-b-D-galactopyanoside (IPTG) was then added and incubation was continued for another 8 h at 16°C to induce protein expression. The bacteria was suspended in ice-cold phosphate-buffered saline (PBS) (pH = 7.4), and homogenized by sonication. The lysate was then centrifuged at 4000 g for 10 min at 4°C. The supernatants were applied to a column containing 0.1 mL of sepharose 4B-glutathione (AmershamP^Q6 ^Pharmacia Biotech). The column was washed with 10 column volumes of PBS buffer. An equal amount of either GST or GST-chCypA (1 mg) bound to sepharose 4B-glutathione was mixed with 1 mg of purified his-M1 protein or 100 μg of MDCK cell lysate infected with influenza A virus, and incubated for 2 h at 4°C. The beads were washed five times with washing buffer (1% NP40, 300 mM NaCl, 20 mM Hepes PH 7.4, 10% Glycerol, 1 mM EDTA) with protease inhibitor cocktail (Roche). Proteins bound to the beads were recovered by adding 2× SDS loading buffer, boiled for 5 min and then analyzed by SDS-PAGE. Proteins were then detected by Western blot with anti-His-tag monoclonal antibody and anti-M1 monoclonal antibody. To perform co-immunoprecipitation, cells transient expression Myc-chCypA and FLAG-M1 proteins were lysed in immunoprecipitation buffer, containing 0.5% NP40, 150 mM NaCl, 20 mM Hepes (PH 7.4), 10% Glycerol, 1 mM EDTA with protease inhibitor cocktail. After centrifugation, the supernatant was incubated with an anti-FLAG antibody (M2; Sigma) for 2 h. Immune complexes were recovered by adsorption to protein G-Sepharose resin (Amersham Biosciences). After five times washes in immunoprecipitation buffer, the immunoprecipitates were analyzed by Western blot.

### Comparing intensity of bands on Western blot X-ray films carried out with Photoshop program

The X-ray film of Western blot was scanned and saved as a grayscale image with resolution to a medium value 500 dpi. Image was shown in Photoshop under *Image > Mode *without color information. Invertion of the dark parts and light parts in image was carried out under *Image > Adjustments*. In this condition, the high-expression bands will have high numerical values when measured. The area of band was selected by drawing a line around the edge of the band with lasso tool. The histogram information of the band including a "Mean" value and a "Pixels" value was recorded for the area within your selection. Bands with high expression are typically darker, but also often larger in size. The values for all bands were entered in a spreadsheet. An integrated measure of the intensity and size of the band was indicated by multiplying the "Mean" value and a "Pixels" value for each band. This integrated value was referred to absolute intensity. Absolute intensity of each chCypA band of different tissues was divided by the absolute intensity of that in liver to come up with a relative intensity for each sample band.

### Generation of A/WSN/33 virus with 12 plasmids reverse genetic system

293T/CypA- cells (1 × 10^6^) were transfected with 12 plasmids reverse genetic system in different amounts (0.1 μg pcDNA-PA, others 1 μg per plasmid) plus 4 μg pCMV-Myc or pCMV-Myc-chCypA using transfect reagent lipofectamine 2000 (invitrogen) according to the manufacturer's instructions. Briefly, DNA and transfection reagent were mixed (2.5 μL of lipofectamine 2000 per μg of DNA), incubated at room temperature for 20 min then added to the cells. Six hours later, the DNA-transfection reagent mixture was replaced by Opti-MEM (GIBCO/BRL) containing 0.01% FBS and 2 μg/mL TPCK treated trypsin (sigma). 48 h after transfection, the supernatant was harvested and A/WSN/33 virus titer was measured with plaque assay on MDCK cells.

### Indirect Immunofluorescence analysis

CEF cells were seeded on slides at 1 × 10^4 ^per well. After the indicated treatment, cells were washed with PBS and fixed in ice-cold 4% paraformaldehyde dissolved in PBS. Nonspecific binding was blocked with 4% BSA in PBST (0.5% Triton). Fixed cells were incubated with primary and secondary FITC-labelled (or TRITC-labelled) antibodies as depicted. Cells nuclei were visualized by DAPI staining and individual cells analyzed by confocal fluorescence microscopy.

### Real-time RT-PCR analysis of chCypA mRNA expression

Real time quantitative RT-PCR was performed with the SYBR premix Ex taq (TaKaRa, Japan) on a corbett 6200 real time detection system (Corbett, Australia) to investigate the expression level change of chCypA in CEF cells after influenza virus infection. Two chCypA primers chCypA-F: 5'CAAGACCGAGTGGTTGGACG3 ' chCypA-R: 5'CCGCAGTTGGAAATGGTGATC3 ' were used to amplify a PCR product of 135 bp, β-actin (GenBank accession No. L08165) was chosen as reference gene for internal standardization (primers sequences as follow:

actinF: 5'CACAGATCATGTTTGAGACCTT3 ' actinR: 5'CATCACAATACCAGTGGTACG3', primers M1F: 5'GGCTAAAGACAAGACCAATCCTG3' and M1R: 5'GTCCTCGCTCACTGGGCAC3' were used to amplify an 87 bp fragment of segment 7 of influenza virus genome. The efficiencies of each primer set were calculated from twofold serial dilution curves. The relative amounts of mRNA were calculated by ΔΔC_T _method. The qRT-PCR amplifications were carried out in triplicates in a total volume of 20 μL containing 10 μL SYBR green 2 × premix, cDNA, primers (final concentration of 0.2 μM). The PCR program was 95°C for 30 s followed by 40 cycles of 94°C for 5 s, 60°C for 30 s and dissociation curve analysis of amplification products was performed at the end of each PCR reaction to confirm that only one PCR product was amplified and detected. Each sample was run in triplicate along with the internal control gene. Data analysis of real time PCR was performed with Rotor Gene 6000 series Software (Corbett, Australia) and the relative amounts of mRNA were calculated by ΔΔC_T _method. C_T _difference between chCypA and β-actin called ΔC_T _were calculated to normalize the differences in the amount of total nucleic acid added to cDNA reaction mixture and the efficiency of reverse transcription reactions. The uninfected group was used as the reference sample, called the calibrator. The ΔΔC_T _for each sample was subtracted from the ΔC_T _of the calibrator and the difference was called ΔΔC_T _value, namely the comparative C_T_. The relative expression level of chCypA could be calculated by 2^-ΔΔCT^, and the value stands for an n-fold difference relative to the calibrator.

### Plaque assay

Plaque assays were performed as described previously [7]. MDCK cell monolayer (at a confluent of 100% in 12 well tissue culture plates) was washed with PBS and incubated with different dilutions of virus for 1 h at 37°C. The virus inoculation was removed and washed with PBS. Cell monolayer was then overlaid with medium (DMEM supplemented with 0.8% low-melting-point agarose and 2 μg/mL TPCK-treated trypsin), when agarose medium solidified, cell culture plate converted incubated at 37°C. Visible plaques were counted at 4 days post infection and virus titer was determined. All data was expressed as the mean of three independent experiments.

## Competing interests

The authors declare that they have no competing interests.

## Authors' contributions

CX conceived and designed the experiments. CX, SM performed the experiments. CX, XL and LS performed data analysis. CX and WL wrote the paper. WL supervised CX and reviewed and edited the manuscript. All authors read and approved the final manuscript.
